# Effect of Wet-Ground Silica Fume on High-Strength Steam-Cured Cement Concrete

**DOI:** 10.3390/ma18051105

**Published:** 2025-02-28

**Authors:** Shiheng Wang, Peng Zhao, Yaogang Tian

**Affiliations:** School of Materials Science and Engineering, Chang’an University, Xi’an 710061, China; ygtian@chd.edu.cn

**Keywords:** high-strength steam-cured concrete, silica fume, wet grinding, mechanical properties

## Abstract

In order to improve the dispersion state of silica fume (SF) in cement concrete, accelerate the hydration rate of high-strength steam-cured cement concrete, and reduce production costs. In this paper, SF was made into a wet-ground silica fume (WSF) suspension solution through a wet grinding process and was applied to high-strength steam-cured concrete to replace the SF so as to improve the difficult dispersion of the inner SF and enhance the compressive strength of concrete. The physical and chemical properties of WSF were studied by XRD, SEM, and ZETA potential, and its effects on the mechanical properties, hydration development, and microstructure of cement concrete were studied using XRD, SEM, TG, BET, and NMR. The results show that SF mixed with water is transformed into a kind of suspension solution by wet grinding. After adding WSF, the compressive strength of concrete at 1 d increases when the substitution of WSF increases. Compared with SF-0, the 1 d compressive strength of SF-1 and SF-2 increased by 9.2% and 12.9%. When the WSF substitution was greater than 50%, the compressive strength of concrete did not improve significantly; the 1 d compressive strength of SF-3 and SF-4 is 14.3% and 15.4% higher than SF-0. With the increase in WSF substitution, the porosity of concrete at 1 d decreases, and the structure becomes denser. XRD, TG, and NMR analyses show that WSF can promote the hydration development of cement to form a C-S-H gel. As the amount of WSF substitution increases, its effect on the cement hydration reaction increases first and then tends to be flat.

## 1. Introduction

In high-strength steam-cured concrete, a high volume of silica fume (SF) is usually used as an admixture. On the one hand, SF can replace part of cement in concrete production, reduce the amount of cement in concrete, and reduce production costs. On the other hand, it can delay the release of concrete hydration heat, improve concrete performance, and reduce resource waste and environmental pollution [1,2]; however, SF particles are easily electrostatically adsorbed and agglomerated, making it difficult to disperse in concrete. In addition, the hydration activity of SF is lower than that of cement, and the secondary hydration reaction of agglomerated SF is slower, which has an adverse effect on the properties of cement concrete [3].

In order to improve the dispersibility of SF in high-strength steam-cured concrete, water reducers are usually used. Its polymer structure has multiple functional groups [4], some of which can be adsorbed on the surface of SF [5]; another part of the hydrophobic functional groups can block water, thereby improving the dispersibility of SF in concrete. Related studies have shown that the adsorption behavior of water reducers on SF can be improved by changing the length of water reducer molecules, the length of side chains, and the number of functional groups [6,7,8], which can improve the dispersibility of SF in concrete. However, polycarboxylate superplasticizers (PCEs) are expensive, which would increase the expense of cement concrete but have little effect on the problem of reduced workability of concrete when a high volume of SF is added. This is because SF has a large specific surface area. Large amounts of it will absorb moisture in concrete, reduce the density of concrete, increase internal pores, and cause the mechanical properties and durability of concrete to decrease [3,5]. On the other hand, PCE can adhere to the surface of each particle inside cement concrete, and the adsorption capacity of different binding sites is different [9]. When the amount of PCE is low, it is adsorbed on cement rather than SF. When the water reducer is adsorbed on the surface of C_3_S, it has an adverse effect on cement hydration, thereby affecting the development of early strength of concrete [4,6,7].

Besides the traditional methods, researchers [10,11] prepared SF colloids through a wet grinding process to improve their dispersibility. Wet grinding technology has been widely used in recent years [12,13,14]. By crushing the admixture with a certain hydration ability into small particles, its specific surface area is increased [13], the internal chemical bonds are exposed, and the ability to adsorb Ca^2+^ is increased, thereby improving its hydration activity [15,16,17]. In the preparation process of SF colloids, the dispersant is added in advance and attached to the surface of SF to form stable SF colloids, thereby improving the dispersibility of SF in concrete. However, this type of research usually focuses on nano-SF [18,19,20]. Although the wet grinding process has been applied in the preparation of nano-SF and has achieved beneficial results, providing ideas and the basis for SF modification, the process is only suitable for the dispersion of nano-SF. This is because the particle size of nano-SF is usually less than 100 nm, while the particle size of SF is between 0.1 and 100 μm, resulting in significant differences in the properties of nano-SF and SF. Nano-SF has high hydration activity [21,22,23] that can promote cement hydration [24,25,26], enhance the early strength of cement concrete [27,28,29], and overcome the adverse effects of water reducers on the early strength of concrete [30,31,32]. Meanwhile, the preparation of SF into nano-SF by wet grinding has high requirements for equipment [33,34,35] due to the large specific surface area of nano-SF [24], of which the usage in concrete is limited. Excessive use of nano-SF will cause the workability of concrete to decrease [36,37,38]. At present, there is relatively little research on the wet grinding process for SF, and the relevant technical parameters are not yet clear. It is unknown how to form a stable SF suspension and whether it can be dispersed well in concrete; therefore, it is necessary to develop an efficient and convenient SF modification process so that SF can be used in large quantities in concrete and have good dispersibility.

In this paper, wet grinding was chosen to prepare a kind of SF suspension, of which the particle size distribution was between nano-SF and SF. After wet grinding, the larger particles of SF were crushed, which increased its specific surface area, exposed its internal chemical bonds [30,31,32], and increased its ability to adsorb Ca^2+^, which was beneficial to the secondary hydration of SF. At the same time, the surface energy of the particles was reduced to form a stable suspension, thereby improving the dispersion in concrete. SF was replaced by 0%, 25%, 50%, 75%, and 100% of WSF in concrete that was studied on the effect of strength development of cement concrete. The effect of WSF on hydration products and microstructure was studied using XRD, SEM, TG, BET, and NMR.

## 2. Materials and Methods

### 2.1. Materials

Portland cement (P.O. 42.5), granulated blast furnace slag (GGBS), and silica fume (SF) were purchased from Shehui Cement Co., Ltd. (Baoji, China), the X-ray fluorescence results of which are shown in Table 1. A total of 3 g of sample was pressed into a 25 mm diameter disc with boric acid and tested using a Bruker S8 TIGER (BRUKER, Billerica, MA, USA). 

### 2.2. Sample Preparation

#### 2.2.1. Preparation of WSF

**Figure 1:**
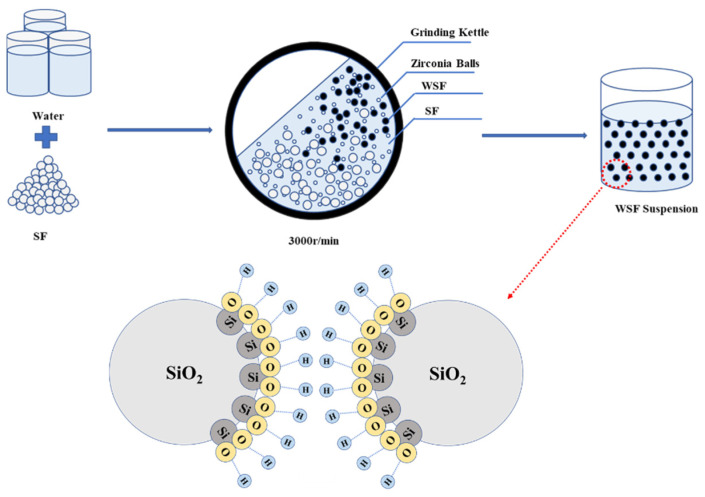
Schematic diagram of WSF.

First, SF and water were combined in a mixture with a ratio of 1:3 (wt%). Then, the mixture was mixed with 1 mm zirconium oxide beads at a ratio of 1:5 (wt%) and wet-milled in a ball mill at 3000 r/min for 120 min to obtain WSF. After wet-milling, the structure of SF was destroyed and the chemical bonds were exposed, which could attract H^+^ hydrolyzed in water and form an H^+^ ion layer around it, making the silica fume particles positively charged. As shown in Figure 1, due to the repulsion between the same charges, the silica fume particles could not agglomerate, thus forming a suspension.

#### 2.2.2. Concrete Sample Preparation

The concrete mix ratio is shown in Table 2. According to the Chinese national standard GB/T 31387-2015 [39], 100 mm × 100 mm × 100 mm concrete specimens were cast and steam-cured for 6 h according to the Chinese national standard GB/T 13476-2023 [40].

### 2.3. Test Methods

**Figure 2:**
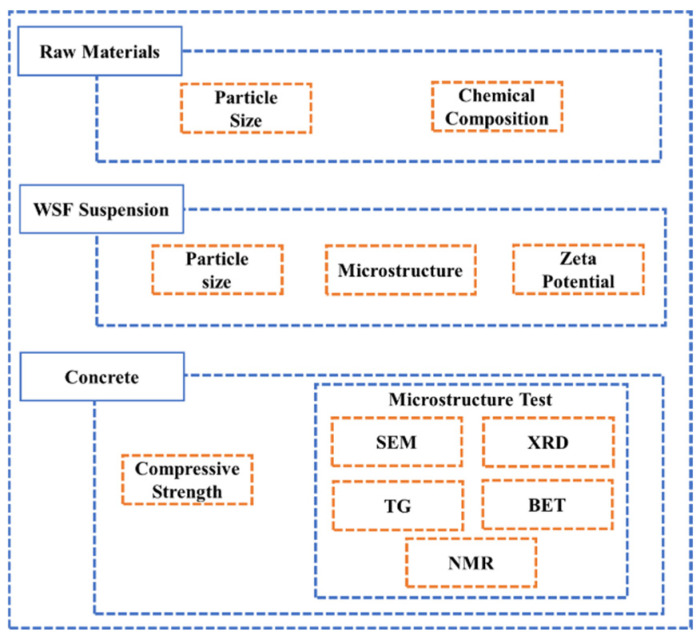
Design of experiments.

The design of experiments applied in the presented work is shown in Figure 2**.**

#### 2.3.1. Particle Size Distribution

The particle size of WSF concrete samples was analyzed using a Malvern Litesizer 500 (Malvern, Worcestershire, UK). The samples before and after wet grinding were dried at 105 °C to constant weight, ground with a mortar to avoid clumping, diluted into a 0.1 g/L solution using a volumetric flask, and ultrasonically dispersed for 5 min. They were tested 3 times, and if the deviation of the test results was greater than 5%, they were retested.

#### 2.3.2. Zeta Potential

The zeta potential of the WSF samples at 0, 30, 60, 90, and 120 min of wet grinding was tested using a Malvern Litesizer 500 (Malvern, Worcestershire, UK) to analyze the degree of hydration during wet grinding. The wet-milled samples were diluted with deionized water and diluted into a 0.1 g/L solution using a volumetric flask, during which ultrasonic dispersion was performed for 5 min. They were tested 3 times, and if the deviation of the test results was greater than 5%, it was retested.

#### 2.3.3. Compressive Strength Test

The compressive strength of WSF concrete samples with replacement of 0%, 25%, 50%, 75%, and 100% was tested using a press machine (TYA-3000, Wuxi Jianyi, Wuxi, China). The WSF concrete was cured for 1 d and 28 d according to GB/T 13476-2023 [40], and three samples were selected from each group of the cured test blocks, and the average value was the result according to GB/T 13476-2023 [40]. They were tested 3 times, and if the deviation of the test results was greater than 5%, they were retested.

#### 2.3.4. XRD Test

The effect of WSF on the hydration characteristics of concrete was analyzed using an X-ray diffractometer (D8 ADVANCE, BRUKER, Billerica, MA, USA). The WSF concrete sample powder was prepared through a 200-mesh screen. The test samples were soaked in ethanol immediately for 24 h to stop hydration and dried at 105 °C to constant weight, ground with a mortar to avoid clumping. They were tested at 4°/min, and the selected test range was from 15° to 60° to analyze the hydration products generated by concrete with different WSF substitution amounts.

#### 2.3.5. SEM Test

The WSF powder and concrete sample powder were observed by scanning electron microscopy (ZEISS SIGMA 360, Carl Zeiss, Oberkochen, Germany) to observe the distribution of their hydration products. A 5 mm × 5 mm thin film WSF concrete sample, which was soaked in ethanol immediately for 24 h to stop hydration and dried at 105 °C to constant weight, was selected. The samples were treated with Pt spraying at an operating voltage of 5 kV.

#### 2.3.6. TG Test

The WSF concrete sample powder was analyzed using a comprehensive thermal analyzer (DSC 3+, METTLER TOLEDO, Zurich, Switzerland). The WSF concrete sample powder, prepared through a 200-mesh screen, was soaked in ethanol immediately for 24 h to stop hydration and dried at 105 °C to constant weight and ground with a mortar to avoid clumping. During the test, nitrogen was used as a protection gas; nitrogen has a protection range of 25 mL/min. The mass change caused by the thermal decomposition of C-S-H gel, CH, etc., at different temperatures, was used to analyze the proportion of hydration products in different WSF concretes. The heating rate was 10 °C/min, and the test temperature was selected to be from 50~800 °C.

#### 2.3.7. BET Test

Using the gas adsorption method, the BET surface area and pore size analyzer (ASAP2020, Micromeritics, Norcross, GA, USA) was used to measure the adsorption of test gas by concrete pores to calculate the size of concrete pores. The WSF concrete sample powder prepared with diameters less than 1mm was the test samples were soaked in ethanol immediately for 24 h to stop hydration, dried at 105 °C to constant weight, and ground with a mortar to avoid clumping. At the same time, the effect of WSF on the internal structure of high-strength steam-cured concrete was analyzed.

#### 2.3.8. NMR Test

The nuclear magnetic resonance (NMR) spectra of concrete with different WSF dosages were measured using a 29Si solid-state NMR spectrometer (JNM-ECZ400S, JEOL, Tokyo, Japan). The WSF concrete sample powder, prepared through a 200-mesh screen, was used as the test samples and soaked in ethanol immediately for 24 h to stop hydration, dried at 105 °C to constant weight, and ground with a mortar to avoid clumping. A sufficient sample was compacted in a container, and the effect of WSF on the hydration degree of high-strength steam-cured concrete was analyzed.

## 3. Results and Discussion

### 3.1. Particle Size Analysis of WSF

**Figure 3:**
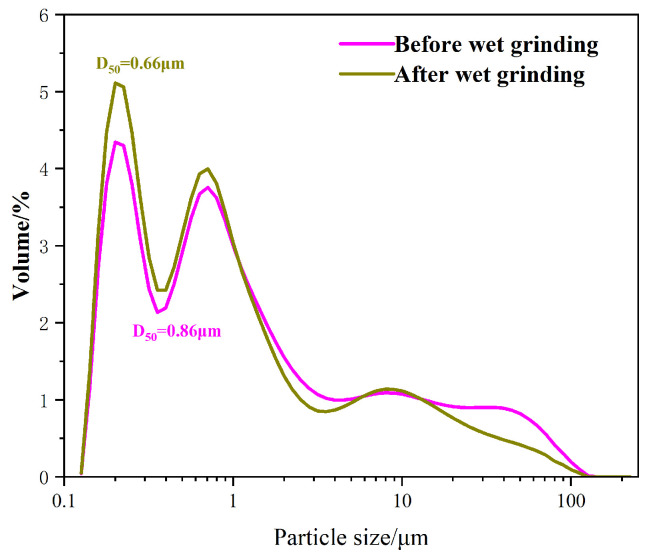
Particle size analysis of WSF.

Figure 3 shows the particle size distribution of WSF; it can be clearly seen that the particle size range of silica fume is 0.1~100 μm, and the D_50_ of silica fume is 0.86 μm. The particle size of WSF decreases after wet grinding. Among them, the particles of 10~100 μm in size decrease, the particles of 0.1~1 μm in size increase, and the D_50_ reaches below 0.66 μm, which is 23.3% smaller than it used to be. The particle size range of WSF particles is more concentrated, indicating that the wet grinding process has a significant effect on reducing the fineness of silica fume and increasing its specific surface area. This helps WSF adsorb Ca^2+^ and improve its secondary hydration ability, thereby accelerating cement hydration.

**Figure 4:**
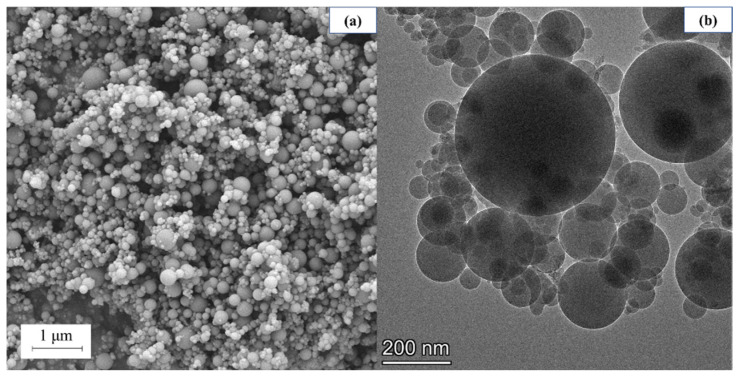
(**a**) SEM morphology of SF and (**b**) TEM morphology of SF.

**Figure 5:**
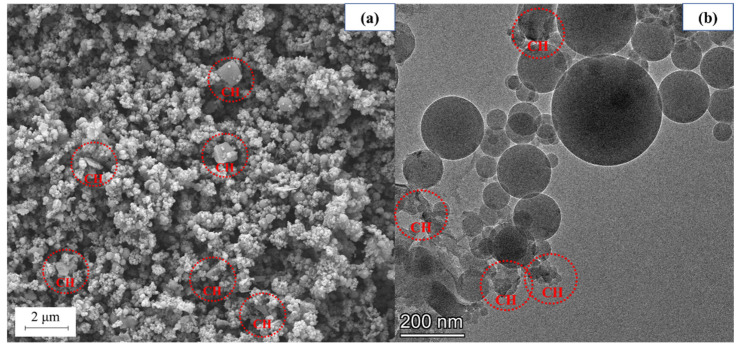
(**a**) SEM morphology of WSF and (**b**) TEM morphology of WSF.

The SEM and TEM morphology of SF is shown in Figure 4, in which the SF ball can be seen. It can be seen that some of the larger particles of SF after wet grinding are broken in Figure 5. At the same time, a low volume of hydration products of SF is found in the SEM image. This is because SF contains 1.46% CaO and 91.08% SF, in which the CaO would react with water during wet grinding to generate CH [40]. CH usually reacts with SF to generate a C-S-H gel, but due to the poor hydration activity of SF and the small amount of CH generated by CaO, it is difficult to observe the C-S-H gel [41]. Figure 5b is a TEM image of WSF. Using TEM, nano-scale flake CH can be clearly observed. Studies have shown that nano-CH helps to adsorb Ca^2+^ generated by cement hydration, thereby accelerating cement hydration. WSF can generate a small amount of nano-CH, which improves the hydration activity of WSF. WSF can accelerate the hydration of concrete.

### 3.2. Zeta Potential Analysis

As the wet grinding time increases, SF undergoes a small amount of hydration reaction. The structure of SF is destroyed, chemical bonds are exposed, and it can attract H^+^ hydrolyzed in water and form an H^+^ ion layer around it, which makes the SF particles positively charged. Due to the repulsion between the same charges, SF particles cannot agglomerate and precipitate, thus forming a suspension [42,43]. However, the hydration reaction of SF is relatively weak and cannot be directly observed using XRD and TG. By analyzing the change in zeta potential, the change in the surface state of SF during wet grinding can be reflected. This is because WSF can attract H^+^ in water and thus change the zeta potential on its surface. The increase in zeta potential makes WSF suspension stable.

**Figure 6:**
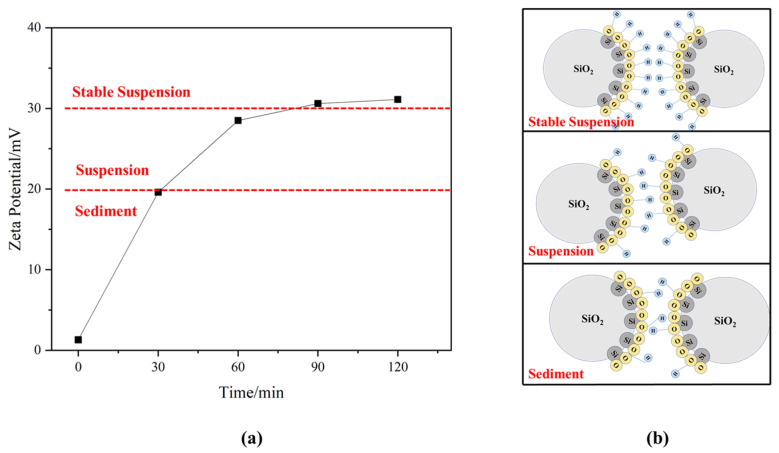
(**a**) Effect of wet grinding on zeta potential of WSF. (**b**) Diagrammatic sketch of zeta potential of WSF.

In Figure 6, the effect of wet grinding time on the zeta potential of WSF is shown. As shown in Figure 6a, the zeta potential of the WSF surface without wet grinding is 1.3 mV, and SF is precipitated in water. After 30 min of wet grinding, the zeta potential of WSF increases to 11.6 mV. When WSF is wet ground for 60 min, the zeta potential increases to 38.5 mV, and a relatively stable suspension can be formed. Subsequently, the growth of the zeta potential tends to be flat because the zeta potential of WSF mainly comes from electrostatic repulsion. As SF is continuously wet ground, its structure is destroyed, and it can attract H^+^ hydrolyzed in water. However, as the degree of wet grinding of SF reaches the maximum, it cannot provide new chemical bonds to attract H^+^, so its zeta potential no longer increases.

### 3.3. Compressive Strength Analysis

**Figure 7:**
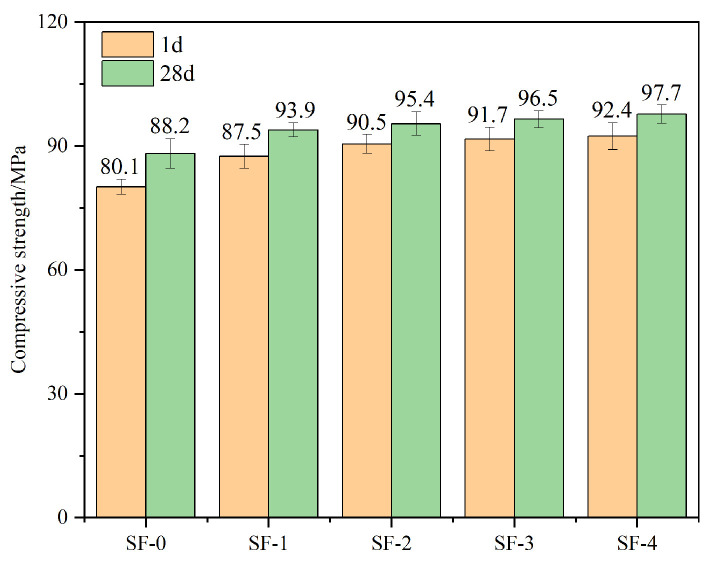
Compressive strength of concrete with different SF content.

Figure 7 shows the effect of WSF on the 1 d and 28 d compressive strength of high-strength steam-cured concrete under different proportions. Compared with SF-0, the 1 d compressive strength from SF-1 to SF-4 is significantly improved and increases with the increase in the WSF nucleus content. The 1 d compressive strength of the SF-1 sample, compared with SF-0, increased from 80.1 MPa to 87.5 MPa with an increase of 9.2%. As the replacement amount of WSF gains, the growth trend of concrete strength slows down. The compressive strength of the SF-2 sample is 90.4 MPa, and the compressive strength of the SF-4 sample is 92.4 MPa, which is an increase of 2.2%. Related studies have shown that by grinding SF to prepare it into nanomaterials, it can be used as a concrete early strength agent to promote cement hydration [24,25]. This is because nano silica fume has a large specific surface area that can absorb a large amount of Ca^2+^, thereby accelerating the dissolution of cement particles and accelerating cement hydration.

Although the particle size of WSF is greater than 100 nm and the specific surface area is smaller than that of nano-SF, it still has a certain ability to adsorb Ca^2+^, which improves its effect in promoting cement hydration. The 1 d compressive strength of concrete added with WSF is higher than that of SF-0, which shows that WSF has a good hydration-promoting effect [11]. When the WSF replacement exceeds 50%, the growth trend of the compressive strength of concrete slows down, but the compressive strength of WSF concrete is higher than that of SF-0. This is because WSF acts as an early strength agent in concrete. When the volume of WSF replacement is low, although it helps to accelerate cement hydration, it can only help the hydration development of part of the cement in the concrete. As the WSF content increases, the cement hydration in the concrete is fully promoted. However, when the WSF content exceeds 50%, the promoting effect on cement is no longer obvious. The compressive strength of high-strength steam-cured concrete cured for 28 d has increased, but the strength of WSF concrete has increased less, all by less than 5%. The compressive strength of SF-0 increased by 11.2%, and the degree of hydration of SF-0 at 1 d was lower than that of concrete with WSF. In the subsequent curing, as the cementitious material is gradually hydrated, the compressive strength increased significantly; however, the concrete with WSF had relatively sufficient hydration at 1 d, resulting in less strength growth in the later period. At the same time, WSF has better dispersibility and can fill the pores of inner concrete better than SF-0, improving the concrete structure and thus increasing the strength of concrete. Therefore, WSF has a comprehensive effect on the compressive strength of concrete through the induced hydration effect and filling effect of C-S-H crystal nuclei and the pozzolanic effect of SF [2,10,11].

### 3.4. XRD Analysis

**Figure 8:**
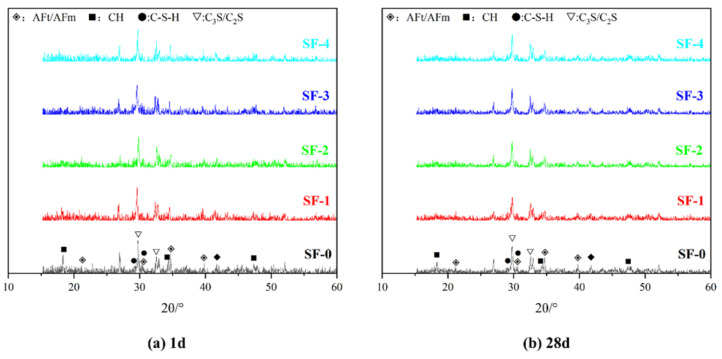
XRD patterns of concrete with different WSF content.

Figure 8 shows the XRD spectrum of concrete with WSF content. In concrete research, the hydration degree of cement is usually analyzed using the CH content because CH is one of the only products of cement hydration [1,2]. The stronger the CH peak, the more hydration products are generated and the higher the hydration degree. However, in this study, the CH generated by cement hydration is affected by the secondary reaction with WSF. The higher the hydration degree of concrete, the less the CH content. As shown in the figure, the characteristic peaks of CH appear at 18.0° and 34.1°, indicating that the CH crystal is in the [001] and [100] crystal orientations at this time. The CH peak of SF-1 at 1 d is significantly lower than that of SF-0. This indicates that the growth of CH crystals is affected. From SF-0 to SF-4, the characteristic peak width of CH at 18.0° narrows, and the peak value decreases to invisible. This suggests that with the SWF application, the average grain size of CH decreases, which indicates that the addition of 25% WSF accelerates the hydration process of concrete, and WSF consumes a large amount of CH. Since the hydration activity of SF in SF-0 is low, the reaction degree with CH is not high, resulting in a large amount of CH remaining. When the WSF content exceeds 50%, the peak intensity of CH is at an extremely low level, indicating that CH is almost completely consumed. At this time, there is no significant difference between SF-2 and SF-4 in the XRD spectrum, indicating that the hydration degree of cement is the same after the WSF content exceeds 50% [17]. As can be seen from Figure 8b, the XRD of concrete at 28 d shows that the characteristic peak at 18.0° of CH in SF-0 decreases significantly than the 1 d SF-0 sample, indicating that CH is consumed by SF and that the hydration reaction of SF-0 continues during this period. The results show that WSF causes a high volume of CH and C-S-H by inducing cement hydration. Meanwhile, it also reacts with CH to form a C-S-H gel for the pozzolanic effect, which is considered to be the main source of concrete’s mechanical properties, thereby improving concrete strength [37]. Therefore, in WSF concrete, as the amount of WSF increases, the promotion effect on cement hydration in concrete tends to be saturated. This makes the hydration degree of concrete no longer increase after 1 d, which matches with the mechanical properties analysis.

### 3.5. SEM Analysis

**Figure 9:**
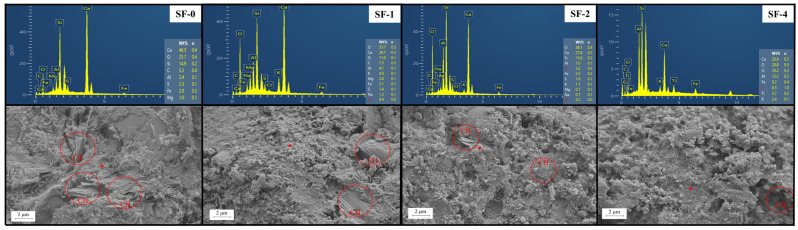
SEM and EDS of concrete with WSF.

Figure 9 shows the SEM and energy dispersive spectrometry (EDS) of steam-cured high-strength concrete for 1 d. There is a large amount of CH in SF-0. As the volume of WSF replaced increases, the CH in concrete gradually decreases. A small amount of CH crystals can be observed in SF-2, but no obvious CH crystals are observed in SF-4. This is because the SF in SF-0 is unevenly dispersed and has low hydration activity, which cannot quickly consume the CH generated by cement hydration [42]. As the amount of WSF replaced increases, the WSF in concrete is more evenly distributed and has higher hydration activity, which can quickly consume CH and generate C-S-H. Therefore, a high volume of network-like C-S-H gel is observed in SF-4.

The Ca^2+^/Si^4+^ ratio is considered to determine the state of the C-S-H gel, and the Ca^2+^/Si^4+^ ratio can be determined by EDS (the red dots in the Figure 9). As the Ca^2+^/Si^4+^ ratio rises, the polymerization degree of the silicon chain in the C-S-H molecule goes down, which causes the C-S-H gel face to change from foil to mesh and fiber. At the end of cement hydration, the C-S-H gel accumulates into a dense structure, and the Ca^2+^/Si^4+^ decreases at this time [44,45]. The test results show that the Ca^2+^/Si^4+^ decreases from SF-0 to SF-4, the Ca^2+^/Si^4+^ of SF-0 at 1 d is 3.11, and that of CS-4 is 1.04. This shows that after the addition of WSF, the C-S-H gel is in a foil morphology, the cement hydration is accelerated, the hydration degree of CS-4 at 1 d is higher than that of SF-0, and it accumulates into a dense structure inside the concrete. The results match with the XRD analysis.

### 3.6. TG Analysis

**Figure 10:**
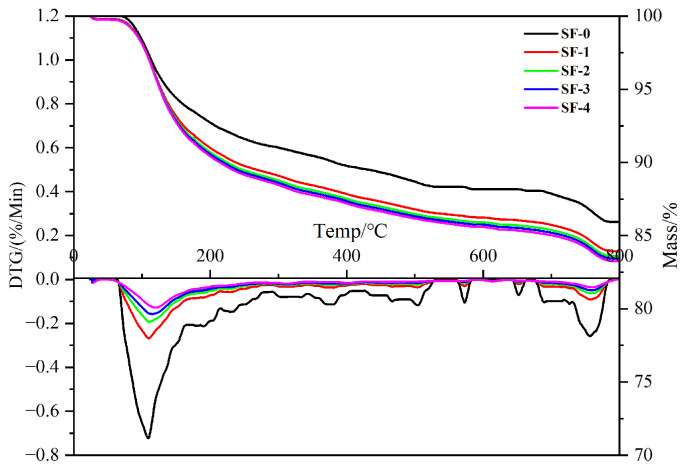
TG of WSF concrete.

Figure 10 is the TG graph of WSF concrete 1 d. Among them, 50–200 °C shows the decomposition of C-S-H gel, Aft/AFm, or water loss. A temperature of 400–500 °C shows the decomposition of CH, and 600–800 °C shows the decomposition of CaCO_3_. As shown in Figure 9, the mass loss of the WSF concrete 1 d sample is significantly increased compared with SF-0, which indicates that more C-S-H gel and Aft/AFm are generated in the concrete. CS-4 loses 10.9% of its mass at 50–200 °C, which is 21.3% higher than SF-0, indicating that more hydration products, such as C-S-H gel, are generated [24,25]. At 400–500 °C, the mass of SF-0 decreases significantly, indicating that it contains more CH. Compared with SF-0, the WSF content of cement with C-S-H gel increases, and CH decreases significantly, which is consistent with the results of XRD. This is because CH in WSF concrete reacts with WSF to form the C-S-H gel for the pozzolanic effect, and its content decreases. The results show that WSF induces cement hydration, consumes a large amount of CH, and undergoes the pozzolanic effect to generate a high volume of C-S-H gel, thereby improving the strength of steam-cured high-strength concrete [27,28]. In different WSF concrete, with the increase in WSF, the growth trend of the C-S-H gel gradually slows down so that the hydration development of concrete increases slowly after 1 d.

### 3.7. Pore Structure Analysis

**Figure 11:**
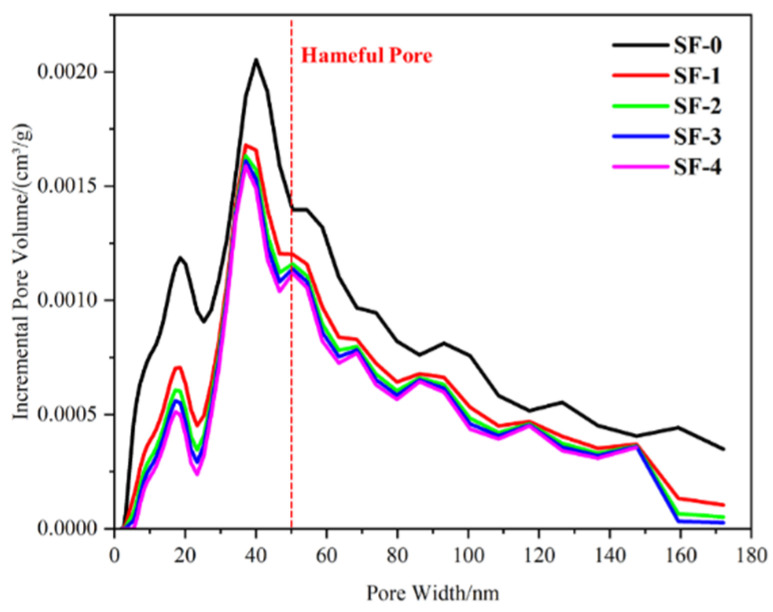
Pore structure in WSF concrete.

Figure 11 is the pore distribution diagram of WSF concrete at 1 d. Generally, the pores in concrete include gel pores (<10 nm) and fine (10–50 nm), medium (50–100 nm), and large capillary pores (>100 nm) [44]. As shown in the figure, WSF can reduce the pore volume of concrete, significantly reduce harmful pores that are larger than 50 nm, and improve the concrete structure [44,45,46]. This is because the nucleation effect of WSF can promote the hydration development of cement to form the C-S-H gel and CH, which fill the concrete and make the concrete structure more compact. At the same time, WSF can react with CH and generate a large amount of C-S-H gel in the pores of concrete, reducing the pore volume and turning the interconnected pores into closed pores [47,48,49]. The good dispersion characteristics of WSF are conducive to the play of the nucleation effect, further reducing the pores of the concrete, which reduces the overall pore volume inside the concrete. It is generally considered that the pore structure of concrete is directly related to compressive strength [49]. Compared with SF-0, the C-S-H content in the WSF concrete increases, while the CH content is greatly reduced, and the structure is more compact, which is consistent with the mechanical properties test. Above all, it is proved that WSF can optimize the pore structure of concrete, provide accelerated cement hydration and secondary hydration of silica fume, and reduce the pore volume of concrete.

Improving the density of concrete can improve its ability to resist the ingress of harmful substances, thereby improving its durability. As shown in Figure 10, the mass loss of SF-0 due to the decomposition of CaCO_3_ in the range of 600–800 °C is 9.4% greater than that of WSF concrete. This shows that SF-0 absorbs more CO_2_ during the preparation and curing process. Concrete carbonation is an important part of concrete durability research [50]. The amount of CO_2_ absorbed reflects the durability of concrete. The denser the concrete, the stronger its resistance to CO_2_. After WSF is added to concrete, the amount of CaCO_3_ decomposed at 600–800 °C decreases, which means that the absorbed CO_2_ decreases, indicating that the interior is denser and has fewer pores. The above results are consistent with the BET test results.

### 3.8. NMR Analysis

**Figure 12:**
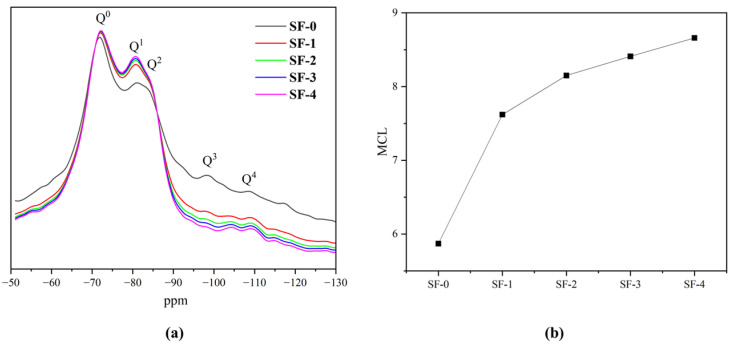
(**a**) NMR graph of WSF concrete and (**b**) MCL graph of WSF concrete.

The NMR spectra of WSF concrete with different additions were studied using ^29^Si NMR. As shown in Figure 12a, Q^n^ represents the chemical bond formed by Si, and n represents the degree of polymerization with Si [45,51]. As shown in the figure, Q^0^ is unhydrated cement concentrated in the area above −75 ppm. The C-S-H gel generated by cement hydration is concentrated in the area between −85 ppm and −75 ppm, represented by Q^1^ and Q^2^; Q^3^ and Q^4^ are the peaks where SiO_2_ is located, usually located in the area between −90 ppm and −110 ppm. The area contained in the Q^n^ peak represents the proportion of the corresponding substance [45]. Figure 10 shows that the integrated areas of Q^3^ and Q^4^ of CS-4 are 37.2% lower than those of the control group. This indicates that WSF reacts with CH generated by cement hydration and is consumed in large quantities. The secondary hydration of WSF generates a high volume of C-S-H gel, making the sum of the integrated areas of Q^1^ and Q^2^ of CS-4 35.2%, which is 55.6% higher than that of the control group.

The main chain length (MCL) of C-S-H gel can be calculated using the following equation:

(1) $$\mathrm{MCL}=\frac{2I\left(Q^{1}\right)+2I\left(Q^{2}\right)}{I\left(Q^{1}\right)}$$

where *I*(*Q^n^*) is the integral of *Q^n^*.

As can be seen from Figure 12b, the MCL of the C-S-H gel increases with the increase in WSF substitution. When the WSF substitution exceeds 50%, the growth of MCL tends to slow down. This shows that the use of WSF can accelerate cement hydration, and through the secondary hydration reaction, CH is consumed, thereby increasing the polymerization degree of the C-S-H gel. When the WSF substitution exceeds 50%, the cement hydration rate in concrete reaches the maximum, and the continued addition of WSF has no significant improvement on cement hydration; it is consistent with the results of XRD and TG tests.

## 4. Conclusions

1. WSF can be prepared using a wet grinding process, and the average particle size of WSF is reduced from 0.86 μm to 0.66 μm. Silica fume undergoes a hydration reaction during wet grinding to produce a low volume of CH and C-S-H gel, thereby increasing the hydration activity of WSF;
2. WSF can improve the 1 d compressive strength of steam-cured high-strength concrete; The 1 d compressive strength gradually enhances with the increasing volume of WSF. When the WSF substitution is greater than 50%, the strength growth is not significant. The 1 d hydration degree of concrete added with WSF is high, which reduces the compressive strength growth after 28 d;
3. WSF can reduce the harmful pores in concrete. WSF can promote the hydration development of cement to form C-S-H gel and CH. CH reacts with SF for a secondary hydration reaction to generate more C-S-H gel, which fills the pores in the concrete and contributes to the denser concrete structure;
4. It can be seen from the XRD, TG, and NMR test results that WSF has a promoting effect on the 1 d strength of steam-cured high-strength concrete. The C-S-H gel content in concrete increases while the CH content decreases, indicating that WSF can promote cement hydration; with the increase in WSF content, the effect of promoting hydration is more obvious; this phenomenon is not obvious when the WSF substitution is greater than 50%.

## Figures and Tables

**Table 1:** Chemical composition of raw materials (wt%).

Type	SiO_2_	Al_2_O_3_	CaO	Fe_2_O_3_	SO_3_	MgO	LOI
Cement	25.64	4.08	60.11	4.01	2.23	2.81	1.12
GGBS	33.75	12.84	41.79	1.31	2.08	7.02	1.21
SF	91.08	0.22	1.46	5.31	0.23	0.91	0.79

**Table 2:** Chemical composition of raw materials (kg/m^3^).

Type	Cement	GGBS	SF	WSF	Fin Aggregate	Coarse Aggregate	Water
SF-0	320	85	40	0	620	1380	110
SF-1	320	85	30	10	620	1380	110
SF-2	320	85	20	20	620	1380	110
SF-3	320	85	10	30	620	1380	110
SF-4	320	85	0	40	620	1380	110

## Data Availability

The original contributions presented in this study are included in the article. Further inquiries can be directed to the corresponding author.
